# Commentary: Trustworthy and ethical AI in digital mental healthcare – wishful thinking or tangible goal?^☆^

**DOI:** 10.1016/j.invent.2025.100844

**Published:** 2025-06-10

**Authors:** Ellen Svensson, Walter Osika, Per Carlbring

**Affiliations:** a Department of Philosophy, Stockholm University, Stockholm, Sweden; b TEA Lab (Trustworthy and Ethical AI Lab), Center for Social Sustainability, Department of Neurobiology, Care Science and Society, Karolinska Institutet, Stockholm, Sweden; c Stockholm Health Care Services, Southern Stockholm Psychiatry District, Region Stockholm, Stockholm, Sweden; d Department of Psychology, Stockholm University, Stockholm, Sweden; e School of Psychology, Korea University, Seoul, South Korea

## Abstract

The use of AI in digital mental healthcare promises to make treatments more effective, accessible, and scalable than ever before. At the same time, the use of AI opens a myriad of ethical concerns, including the lack of transparency, the risk of bias leading to increasing social inequalities, and the risk of responsibility gaps. This raises a crucial question: Can we rely on these systems to deliver care that is both ethical and effective? In attempts to regulate and ensure the safe usage of AI-powered tools, calls to trustworthy AI systems have become central. However, the use of terms such as “trust” and “trustworthiness” risks increasing anthropomorphization of AI systems, attaching human moral activities, such as trust, to artificial systems. In this article, we propose that terms such as “trustworthiness” be used with caution regarding AI and that when used, they should reflect an AI system's ability to consistently demonstrate measurable adherence to ethical principles, such as respect for human autonomy, nonmaleficence, fairness, and transparency. On this approach, trustworthy and ethical AI has the possibility of becoming a tangible goal rather than wishful thinking.

## Highlights

• AI in digital mental healthcare can make treatment more efficient and accessible.
• AI raises ethical concerns regarding transparency, bias, fairness, accountability.
• The EU wants to regulate AI to promote “human-centric and trustworthy AI”.
• Trust and Trustworthiness in AI risks anthropomorphization of these systems.
• Trustworthiness should be consistent and measurable adherence to ethical principles.

In an era of rapid technological innovation (Smith et al., 2023), the promise of AI in digital interventions is transforming our approach to treatment (Egan et al., 2024), but it also raises a crucial question: Can we truly rely on these systems to deliver care that is both ethical and effective? Recent breakthroughs have not only expanded the reach of digital interventions but also sparked debates about the reliability and ethical implications of relying on AI in such a sensitive field (Carlbring et al., 2023). A few years ago, this discussion would have seemed absurd. But, in recent years, AI-powered digital mental healthcare has shown increasingly positive results due to its effectiveness, accessibility, and scalability in the mental healthcare sector (Bolpagni et al., 2024; Pan et al., 2024). AI-based treatments are proving to be valuable complements to in-person care, showing promising results in treating conditions ranging from depression to social anxiety disorder (SAD) and substance abuse (Fu et al., 2020; Gutierrez et al., 2024; Schäfer et al., 2024). Aside from its effectiveness, AI-powered digital mental healthcare promises to make mental healthcare more accessible to socioeconomically disadvantaged groups. In the US and Canada, one in five adults experience mental illness, yet less than 50 % have access to treatments. Globally, approximately 70 % of the world's population receives no formal treatment for their mental health symptoms (Gutierrez et al., 2024). Considering the social and economic burden, together with the individual suffering caused by mental health illness, there is a critical need for increased accessibility to treatment. This became even more evident during the COVID-19 pandemic, when service gaps widened dramatically, underscoring the critical need for accessible mental health services. AI-powered mental healthcare presents the opportunity to make these treatments more accessible, both physically and financially, in addition to lowering the bar for seeking help for certain symptoms. At the same time, the introduction of AI in the mental healthcare sector raises a myriad of ethical considerations and questions (Jain et al., 2024). The opaque nature of AI systems and lack of explainability for certain outputs, risk leading to epistemic injustices, where patients and clinicians may struggle to understand and question the reason for a certain output. In addition, there are issues regarding biases and fairness in mental health diagnosis and treatments, that may increase existing social inequalities. Another central concern regards responsibility gaps and the lack of accountability in AI decisions, as AI-powered mental health interventions risk lacking traceability. Simply put, there is no clear accountability when AI systems provide a harmful or incorrect diagnosis (Mittelstadt et al., 2016; Morley et al., 2020). In the attempt to regulate and ensure the safe usage and application of AI-powered tools, calls for trustworthy AI systems have become central. In 2019, the European Commission's High-level Expert Group on AI (HLEG) argued that we should establish a relationship of trust with AI to cultivate trustworthy AI (Ethics Guidelines for Trustworthy AI, 2019). The AI HLEG guidelines state seven key requirements for Trustworthy AI: (1) human agency and oversight, (2) technical robustness and safety, (3) privacy and data governance, (4) transparency, (5) diversity, non-discrimination and fairness, (6) environmental and societal wellbeing and (7) accountability. Importantly, these ethical principles are only a part of trustworthiness which, according to the EU guidelines, also require legal compliance and technical robustness throughout the system's life cycle. In the EU AI Act from 2024, the first chapter also states that the purpose of the regulation is to promote “human-centric and trustworthy artificial intelligence” (EU AI Act Ch. 1 Art. 1) At the same time, efforts to develop “trustworthy AI” have been criticized for understanding trustworthiness in terms of the acceptability of risks (Aboy et al., 2024; Laux et al., 2024). We propose that terms such as “trustworthiness” be used with caution regarding AI and that when used, they should reflect an AI system's ability to consistently demonstrate measurable adherence to ethical principles, such as respect for human autonomy, nonmaleficence, fairness, and transparency. These principles coincide with the EU requirements, since there is a clear overlap between the existing definition according to EU HLEG and what we propose here. Key requirement (1) coincides with respect for human autonomy, key requirements (2) and (6) coincide with nonmaleficence, and key requirements (4) and (5) cover transparency and fairness respectively. Our novel contribution is therefore to translate these principles into measurable performance indicators, tailored to digital mental health. As a practical implication, we suggest that clinicians and developers use the EU assessment list for trustworthy AI as a baseline and then add domain-specific metrics (Löchner et al., 2025). For example, measures of clinical safety, patient autonomy in treatment choice, and continuous fairness audits, to operationalize our four core ethical principles within mental health. This, we argue, does not require a new definition of trustworthiness concerning AI systems, but rather a grounding of the concept in widely accepted ethical principles for the use of AI. Still, this raises the question of whether trust and trustworthiness are the right approach to AI systems. It remains unclear whether AI could even be considered a genuine object of trust due to the lack of human qualities like intentionality and agency (Laux et al., 2024; Nickel et al., 2010; Ryan, 2020; Weydner-Volkmann and Feiten, 2021). Some have argued that the discussion of trust risks clouding our judgments in relation to AI since this anthropomorphization attaches human moral activities, such as trust, to artificial systems. On this view, AI can only be considered reliable but never trustworthy, due to lacking emotions and affection towards other parties in addition to not being responsible for its own actions (Ryan, 2020). Simply put: AI systems are not humans and trust is a human activity between parties that can feel for each other and be held accountable to each other. Thus, the question becomes not only whether we can trust AI systems, but also whether trust is the appropriate metric, or if this focus risks missing the target, in evaluating their role in mental healthcare. That is, if the goal is to create AI systems that can adhere to bioethical principles and current AI legislation such as the EU AI Act (2024) (Aboy et al., 2024; Brodersen et al., 2025) is trust what we should strive towards? If not, then this would entail a completely different approach to mental healthcare than what has traditionally been practiced. Trust is both a central pillar to the patient-doctor relationship as well as being considered necessary for basing a decision on a certain output (O'Neill, 2002). If the approach to the use of AI in digital mental healthcare is not to be a trusting one, then what approach towards the AI systems ought patients and clinicians have? At the same time, the trust-based relationship between patients and clinicians has previously led to a hierarchical setting where the patients must place an excessive amount of trust in their healthcare givers, often leading to paternalistic practices in medicine. In the same way, excessive trust in AI systems providing mental health care could lead to new forms of paternalism, where algorithmic decision-making overrides patient autonomy, in the shape of AI paternalism (Kühler, 2022; Voinea et al., 2024). So, where does this leave us? One option is to abandon the concept of trust in regard to AI altogether. This seems a drastic choice, to say the least. Seeing that trust plays a fundamental role in both decision-making and care relationships, such as doctor-patient and therapist-patient relationships, abandoning trust as a concept would imply a complete reevaluation of our understanding of healthcare at large. A more tangible goal is to redefine trust and trustworthiness in AI systems in a way that does not require anthropomorphization, assigning human-like emotions and intentions to technology, but instead measures these systems solely on their adherence to ethical principles, including respect for human autonomy, nonmaleficence, fairness, and transparency. In this view, trustworthiness relates to an AI system's performance in different stages of its lifecycle, and the trustworthiness of an AI system can be evaluated with respect to the tensions between the ethical principles during the different stages of the AI lifecycle (Vetter et al., 2023; Zicari et al., 2021a, Zicari et al., 2021b). From this perspective, trustworthy and ethical AI has the possibility of becoming a tangible goal rather than wishful thinking. As we stand, the emerging breakthroughs in AI and robotics have the possibility to revolutionize mental healthcare, transforming these systems from mere diagnostic screening tools into empathetic partners able to echo the support provided by human caregivers. At the same time, it is of utmost importance that we do not outsource important personal decisions and procedures to AI systems that we do not fully understand and should not fully trust. If trustworthiness is to be understood as consistent and measurable adherence to ethical principles such as respect for human autonomy, nonmaleficence, fairness, and transparency, these principles must be made applicable and tangible in relation to AI-integrated mental healthcare. We may not need to redefine trustworthiness, but further research is needed to understand how trust in AI systems may change over time if AI-powered online mental healthcare becomes widespread.

## Funding

This research did not receive any specific grant from funding agencies in the public, commercial, or not-for-profit sectors.

## Declaration of competing interest

The authors declare that they have no known competing financial interests or personal relationships that could have appeared to influence the work reported in this paper.
